# Self-Assembly of Amyloid Fibrils into 3D Gel Clusters versus 2D Sheets

**DOI:** 10.3390/biom13020230

**Published:** 2023-01-24

**Authors:** Kanchana Karunarathne, Nabila Bushra, Olivia Williams, Imad Raza, Laura Tirado, Diane Fakhre, Fadia Fakhre, Martin Muschol

**Affiliations:** Department of Physics, University of South Florida, Tampa, FL 33620, USA

**Keywords:** hen egg-white lysozyme, amyloid fibril, self-assembly, gelation, plaque formation

## Abstract

The deposition of dense fibril plaques represents the pathological hallmark for a multitude of human disorders, including many neurodegenerative diseases. Fibril plaques are predominately composed of amyloid fibrils, characterized by their underlying cross beta-sheet architecture. Research into the mechanisms of amyloid formation has mostly focused on characterizing and modeling the growth of individual fibrils and associated oligomers from their monomeric precursors. Much less is known about the mechanisms causing individual fibrils to assemble into ordered fibrillar suprastructures. Elucidating the mechanisms regulating this “secondary” self-assembly into distinct suprastructures is important for understanding how individual protein fibrils form the prominent macroscopic plaques observed in disease. Whether and how amyloid fibrils assemble into either 2D or 3D supramolecular structures also relates to ongoing efforts on using amyloid fibrils as substrates or scaffolds for self-assembling functional biomaterials. Here, we investigated the conditions under which preformed amyloid fibrils of a lysozyme assemble into larger superstructures as a function of charge screening or pH. Fibrils either assembled into three-dimensional gel clusters or two-dimensional fibril sheets. The latter displayed optical birefringence, diagnostic of amyloid plaques. We presume that pH and salt modulate fibril charge repulsion, which allows anisotropic fibril–fibril attraction to emerge and drive the transition from 3D to 2D fibril self-assembly.

## 1. Introduction

Amyloidoses refer to a series of human disorders characterized by the deposition of protein plaques, i.e., dense aggregates of protein fibrils in the affected tissues and organs [1,2,3,4]. While the proteins composing the fibrils are disease-specific, the fibrils themselves all share a cross-beta sheet core structure [3,5,6]. This structural commonality has been linked to the underlying propensity of polypeptide chains to form intermolecular hydrogen bonds across their backbone [5,7]. In addition, fibril plaques display apple-green birefringence upon staining with Congo Red, indicative of a high degree of alignment of the individual fibrils within the plaques. The role of amyloid fibrils as the underlying cause of disease symptoms remains controversial. There is compelling evidence that small oligomers, emerging during the assembly of amyloid fibrils and plaques, are critical contributors to proteotoxicity, particularly in neurodegenerative diseases [8,9,10,11,12,13,14]. At the same time, the neurotoxicity of poly-Q fibrils in Huntington’s disease was shown to decrease upon fibrils forming extended bundles. In addition, the significant accumulation of dense fibril deposits and resulting disruption of tissue structures in non-neuropathic amyloidoses are considered the primary causes of disease pathology [15,16]. Much experimental and theoretical progress has been made in characterizing and modeling details of the structure and growth kinetics of individual fibrils from monomeric proteins [17,18,19,20,21,22,23,24,25]. In comparison, the process of self-assembly of individual amyloid fibrils into large plaques in vitro has received relatively little attention [26,27]. Similarly, the origin and structure of diffusive vs. dense-core amyloid plaques in vivo, observed at different time points in Alzheimer’s Disease and Down Syndrome, remains unresolved [28,29]. Recently, it was even suggested that the formation of dense-core plaques is not a spontaneous process, but requires active assembly by microglia [30]. 

At the same time, multiple proteins have been shown to form non-toxic functional amyloid fibrils. Their physiological functions range from antimicrobial activity and peptide storage to spermatozoa selection and clearance to, perhaps most surprisingly, memory consolidation [31,32,33,34]. This, combined with their attractive material properties, their stability and their ability to self-assemble, has made amyloid fibrils an attractive candidate as substrates or scaffolding for creating novel biomaterials [35,36]. This includes, in particular, the ability of amyloid fibrils to form 3D scaffolds for cell adhesion [37,38]. Hence, understanding the mechanisms regulating the self-assembly of amyloid fibrils into various ordered suprastructures relates to our understanding of the origin of various amyloid plaques observed in vivo, as well as to our ability to control and direct the self-assembly of fibrils into distinct mesoscopic and macroscopic suprastructures for use as biomaterials. 

We wanted to study the intrinsic self-assembly behavior of fibrils and the types of fibrillar suprastructures they form upon changes in ionic strength or pH of their solution environment. Changing the ionic strength, we explored the effects of charge screening on fibril self-assembly. Changing the pH, in turn, altered the radial and, consequently, axial charge distribution of the fibrils. In most studies of supramolecular fibril assembly, though, experiments grow fibrils and their resulting superstructures starting from monomers [38,39,40]. This has several drawbacks. For one, fibril growth tends to generate toxic oligomers and protofibrils that are undesirable in biomaterials. Furthermore, there is evidence that both of these latter aggregate species directly interact with and alter fibril assembly [41]. In addition, the growth behavior and relative distribution of these various amyloid aggregate species, themselves, is a sensitive function of protein concentration and growth conditions [42]. Therefore, any observed changes in fibrillar suprastructures resulting from changes in solution conditions would be a composite of the intrinsic behavior of fibrils themselves, as well as changes in the overall composition of amyloid aggregate species emerging under different growth conditions. We therefore separated the fibril growth process from subsequent fibril self-assembly by using preformed lysozyme fibrils. In addition, we used fibril growth conditions we had previously identified for minimizing the formation of oligomeric off-pathway intermediates [42].

## 2. Materials and Methods

### 2.1. Protein and Chemicals

Two times recrystallized, dialyzed and lyophilized hen egg white lysozyme (hewL) was purchased from Worthington Biochemicals (Lakewood, NJ, USA). Ultrapure grade Thioflavin T (ThT) was obtained from Anaspec (Freemont, CA, USA). Other chemicals were from Fisher Scientific (Pittsburgh, PA, USA) and were reagent grade or better. All solutions were prepared using 18 MΩ water from a reverse osmosis unit (Barnstead E-pure, Dubuque, IA, USA). 

### 2.2. Growth and Separation of Lysozyme Amyloid Fibrils

Fibrils were grown at 1.4 mM lysozyme concentration with 50 mM NaCl at pH 2 in either 25 mM KH_2_PO_4_ buffer or in water. In either case, HCl was used to adjust the final solution pH to 2.0. The water-based fibril stock was used for transferring fibrils to pH 7. All the buffers/solutions were freshly prepared and filtered through 220 nm pore size syringe filters. The resulting HEWL solutions were placed in a heat bath at 42 °C for 1–2 min and were subsequently filtered through 220 nm and 50 nm syringe filters. Final HEWL concentration was determined from their UV absorption at 280 nm (ε_280_ = 38,940 cm^−1^ M^−1^) using a UV spectrophotometer (Denovix DS-11). Typically, 20 mL of HEWL solutions were prepared, divided among six 15 mL conical centrifuge tubes (Thermo Fisher Scientific) with a volume of 3–4 mL each and incubated at 52 °C for 5–6 days. Following incubation, dynamic light scattering (DLS) was used to confirm the formation of fibrils. Fibrils were isolated from monomers and any potential oligomer background using three repeated centrifugation steps. In each step, fibrils solutions were spun at 14,000 *g* and 15 °C for 24 h. The resulting fibril pellet was collected and resuspended in 50 mM NaCl with 25 mM KH_2_PO_4_ buffer or with pH-adjusted water, while the supernatant was discarded. After the 3rd spin, the resulting HEWL fibril pellets were resuspended in 0 mM NaCl and either 25 mM KH_2_PO_4_ at pH 2 or pH adjusted water to final (monomer-equivalent) concentrations of 40–100 µM. This fibril stock was stored at 4 °C for further experiments. The morphology of the fibril stock was determined using transmission electron microscopy (TEM). The high-speed centrifugation (14,000 *g*) used for fibril isolation tended to induce a few isolated plaques. Therefore, before conducting fibril aggregation experiments, we spun the isolated fibril solution at 6000 *g* for 5 min to sediment the small plaques and used the pure fibril supernatant. 

### 2.3. Salt Mediated Fibril Assembly at pH 2 

A total of 20 µM of isolated HEWL fibril were suspended in 25 mM KH_2_PO_4_ pH 2 buffers with NaCl concentrations ranging from 100 to 400 mM. Solutions were prepared by mixing protein and salt stock solutions at a 1:1 ratio, each at twice their final concentration. The desired series of NaCl concentrations were achieved by diluting 2 M NaCl with 0 M NaCl, both buffered with 25 mM KH_2_PO_4_ at pH 2. All buffers were filtered through a 220 nm syringe filter before mixing with the HEWL fibril stock. Due to the rapid aggregation of fibrils at higher salt concentration, the concentration of the 2× fibril stock was measured prior to mixing with NaCl. Due to rapid precipitation after mixing, the concentration of the 2× fibril stock, divided by 2, was used as the initial protein concentration.

### 2.4. Visualization of Aggregate Morphology using Fluorescence Microscopy

To document aggregate morphologies, 20–30 µL of a given solution were stained with 15–50 µM ThT. ThT stock solutions were prepared by dissolving the dye in distilled water and filtering it through a 220 nm pore size Nylon syringe filter. Thioflavin T concentration was determined from absorption at 412 nm (α_412_ = 26, 620 mL mg^−1^ cm^−1^).

Samples were placed on #1.5 glass slides (Marienfeld superior^TM^, Electron Microscopy Sciences) which had been cleaned by sonicating in 95% Ethyl alcohol for 5–10 min and dried using nitrogen. To prevent sample evaporation while also preserving the 3-D structures of gel clusters, a white reinforcement label ring was placed on the glass slide. Then, 1.5 µL of sample was deposited in the middle of the ring and the droplet was covered with a pre-cleaned coverslip. Images of the droplets were obtained with an inverted fluorescence microscope, (Olympus IX-70) using 10× (Olympus UPlanFl, NA = 0.30) or 40× (Olympus UPlanFl, NA = 0.75) objectives and images collected with an IXON^EM^ + EMCCD camera (Andor Technology). ThT fluorescence was excited with a 455 nm LED (Thorlabs M455L2) and visualized in epifluorescence with a filter cube using a 445/20 nm ex., 458 nm dichroic and 482/35 nm em. filter. 

### 2.5. Quantification of Fibril Aggregation Using Centrifugation

To quantify the degree of fibril assembly under different solution conditions and incubation periods, we used a modified centrifugation protocol. Aggregated fibril solutions (500 μL) were spun at 6000 *g* for 5 min to sediment the large structures. To avoid any re-mixing with the sedimented large structures, 150 µL of supernatant was collected gently from the top of the centrifuged solution. The concentration of fibrils remaining suspended in the supernatant was determined using UV absorption at 280 nm. Concentrations were measured using a small volume quartz cuvette with 10 mm path length. To minimize errors from instrumental drifts, the spectrophotometer was allowed to warm up for 2 h and prior to each sample measurement, a new buffer background was obtained. Calibration with lysozyme monomers indicated a resolution of 0.02 absorbance units, or equivalent 0.5 μM lysozyme concentration, using this approach.

### 2.6. Salt-Mediated Fibril Assembly after Transfer to pH 7

To transfer fibrils from pH 2 to pH 7, we used isolated fibrils in pH 2 adjusted water. Fibril stock (100 μM) was diluted 5× to a final concentration of 20 μM into 50 mM HEPES buffer at pH 7 and at various salt concentrations. HEPES buffer had a final concentration of 40 mM. The desired salt concentration was achieved by mixing 2 M NaCl with 0 M NaCl solutions at the proper ratio, both buffered with 50 mM HEPES buffer at pH 7.

### 2.7. Dialysis Protocol

Next, 20 μM HEWL fibrils were incubated for 24 h in either 25 mM KH_2_PO_4_ pH 2 buffer and 400 mM NaCl or transferred into 40 mM HEPES pH 7 and 100 mM salt. A total of 500 μL of the resulting gels (pH 2) or plaques (pH 7) were placed in mini dialysis tubes (Pierce, Slid-A-Lyzer, 10 kD, 15 mL tube) and dialyzed against 14 mL of KH_2_PO_4_ buffer at pH 2.0 without salt. Samples were dialyzed for 7 days at room temperature while being agitated with a slow shaker at 35–40 rpm. After 7 days, the protein samples were transferred from the dialysis chamber into centrifuge tubes and the aggregate quantification protocol (see above) was followed to determine the degree of aggregate dissociation back into individual fibrils. Samples were also imaged prior to centrifugation using fluorescence microscopy. 

### 2.8. Transmission Electron Microscopy 

Isolated HEWL fibril samples were diluted into distilled water right before depositing 10 µL onto the surface of Formvar/carbon film coated, 200 mesh copper grids and allowed to air dry. Then, 10 µL of distilled water was added and blotted 3 times to remove salt crystals from the grid. The grid was negatively stained with 10 µL of 8% (*w*/*v*) uranyl acetate for 1–2 min, blotted and air dried. Excess uranyl acetate was removed by repeated washing with distilled water and the grid was left to air dry. All the grids were imaged using a FEI Morgani transmission electron microscope at 60 kV with an Olympus Mega View III camera.

### 2.9. Dynamic Light Scattering (DLS)

Successful fibril growth after incubation was confirmed using dynamic light scattering [43]. A Zetasizer Nano S (Malvern Instruments, Worchestershire, UK) equipped with a 4 mW He-Ne laser (λ = 633 nm) and back scattering geometry (θ = 173°) was used to perform dynamic light scattering (DLS) measurements. Correlation functions were collected over three minutes, and were converted in particle size distributions via the built in deconvolution analysis package in the Zetasizer software, v3.30.

## 3. Results

### 3.1. Growth and Characterization of Isolated Lysozyme Amyloid Fibrils

**Figure 1:**
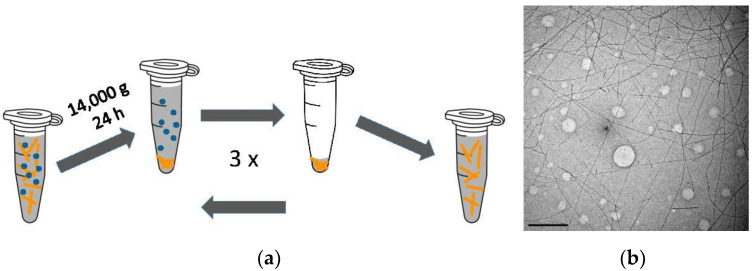
Preparation and Characterization of Isolated hewL Amyloid Fibrils. (**a**) HewL fibrils were pelleted at 14,000 *g* and 15 °C for 24 h. The supernatant was discarded, and the pellet resuspended in buffer or pH adjusted water. This process was repeated 3× to obtain the isolated fibril stock used in all subsequent experiments. (**b**) TEM image of isolated HEWL fibril stock, diluted to 0.2 µM monomer equivalent. Scale bar: 500 nm. (Light circles are due to defects in TEM grid).

We had previously developed a protocol for generating isolated lysozyme amyloid fibrils [44]. Briefly, lysozyme fibrils were grown by dissolving 1.4 mM of hewL monomers in 50 mM NaCl at pH 2, using either 25 mM KH_2_PO_4_ buffer or HCl-adjusted dH_2_O. Solutions were incubated at 3 mL solution volume in 15 mL tubes at T = 52 °C for 5–6 days. These growth conditions reproducibly generate suspensions of individual hewL amyloid fibrils, while minimizing oligomer formation [42,43]. To isolate hewL fibrils from residual monomers and oligomeric admixtures resulting from lysozyme “nicking” [44,45], solutions were subjected to three cycles of a centrifugation and resuspension protocol. In each cycle, solutions were spun for 24 h at 14,000 *g*, with the resulting fibril pellets re-suspended in zero salt pH 2 buffer (Figure 1a). Dynamic light scattering after the last spin indicated the successful removal of non-fibrillar aggregates and monomers from the supernatant. Resuspensions of the fibril pellet provided the stock solutions for subsequent bundling experiments. Transmission electron microscope images of the resulting hewL fibrils display individual rigid fibril of uniform width and of multiple microns in length (Figure 1b). The repeated centrifugation at 14,000 *g* did induce some minor fibril aggregation. These aggregates were removed with a short round of centrifugation for 5 min at 6000 *g*.

### 3.2. Salt-Induced Precipitation of Isolated Amyloid Fibrils at pH 2

Lysozyme monomers at pH 2 carry a net charge of approx. +17, which implies that their corresponding amyloid fibrils are highly positively charged [46,47]. As a result, fibrils in zero-salt solutions remain stably suspended for many weeks. To explore the colloidal stability of these fibril suspensions, we systematically increased the ionic strength of the solutions while keeping fibril concentrations fixed. To do so, we mixed 2× concentrated fibril stock in 0 NaCl and buffer with a 2× concentrated NaCl and buffer solution in equal proportions, resulting in a final fibril concentration of 20 μM (monomer equivalent) and NaCl concentrations ranging from 50 and 400 mM. The final volume for each mixture was 1.5 mL, with 500 µL of each condition incubated in three separate centrifuge tubes. After incubation, ranging from as little as a few minutes to five days, the resulting aggregate structures were visualized with fluorescence microscopy and the overall degree of aggregation was quantified using centrifugation.

**Figure 2:**
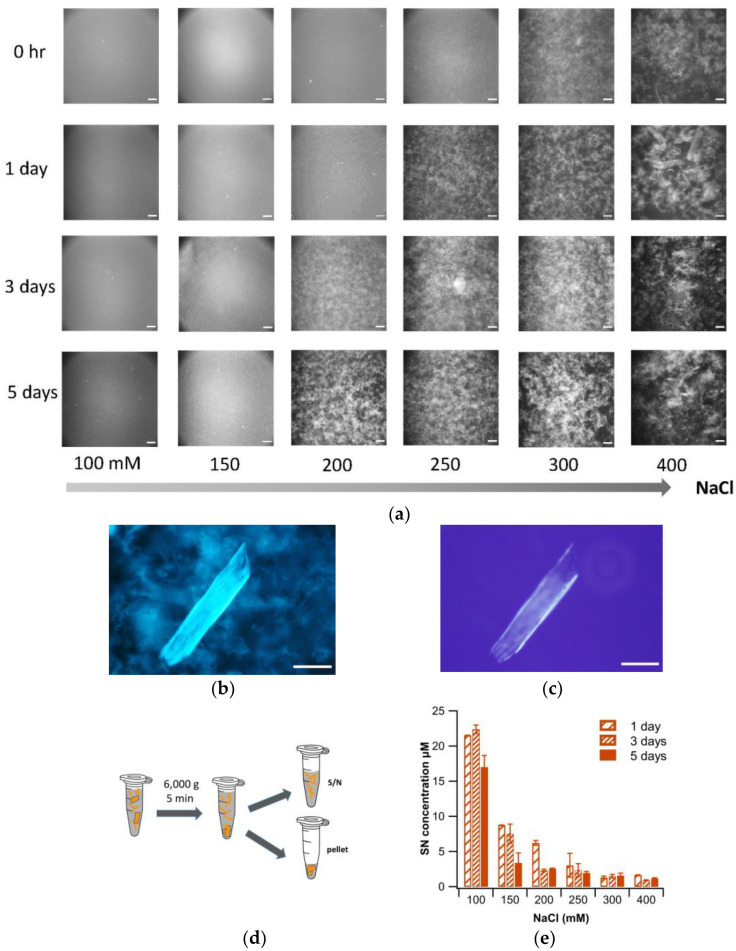
Salt-induced Fibril Gel Clusters at pH 2. (**a**) Fluorescence microscopy images of ThT stained aliquots, imaged at the indicated time points and salt concentrations, and prior to centrifugation assay in (**d**). 3D gel clusters are observed, which increase noticeably in size with salt concentration and incubation period. (**b**,**c**) ThT fluorescence (**b**) and cross-polarization (**c**) image of fibrils incubated at 400 mM NaCl for 5 days. At 400 mM NaCl and extended incubation, 2D sheet-like plaques can form alongside 3D gel clusters. Scale bars in all images: 100 µm. (**d**) Schematic of aggregate/fibril separation assay. (**e**) Quantification of lysozyme fibrils remaining in suspension, i.e., not incorporated into aggregates, following the separation protocol in (**d**). All fibril concentrations were (21 ± 1) µM prior to incubation.

To visualize aggregate morphologies, small aliquots of solution were removed, stained with thioflavin T and imaged using fluorescence microscopy. As shown in Figure 2a, increasing salt concentration beyond 100 mM NaCl caused fibrils to come out of suspension and assemble into low-density three-dimensional gel clusters. The numbers and densities of these clusters increased both with salt concentration and incubation period. At the highest salt concentration used (400 mM), a second, distinct type of supramolecular assembly emerged: dense and ordered 2D sheets (Figure 2b). Besides their bright ThT fluorescence, these sheets tended to display noticeable birefringence under crossed polarizers (Figure 2c), considered a hallmark of amyloid plaques. This birefringence indicates that fibrils became linearly aligned over extended regions within a given fibril sheet. Gel clusters, in turn, did not display any signs of birefringence.

To quantify the extent of fibril aggregation as a function of salt concentration and incubation period, samples were subjected to a modified centrifugation protocol (Figure 2d). Specifically, samples were spun at 6000 *g* for only 5 min, removed immediately from the centrifuge and 150 μL of the supernatant was used to determine the concentration of fibrils remaining suspended. The decrease in the fibril concentration in the supernatant was taken as a quantitative read-out of the increase in aggregates left behind in the pellet. Figure 2e shows the amount of fibrils remaining in suspension following 1, 3 and 5 days incubation at the indicated salt concentrations. The results of the centrifugation data match with the corresponding fluorescence images taken prior to centrifugation. This confirms that the extent of aggregation obtained via the modified centrifugation protocol (Figure 2d) is not affected by the mechanical separation procedure, but reflects the extent of fibril aggregation under these conditions. The data quantify the progressive loss of colloidal stability of the fibrils and resulting gel clustering upon incubation at 150 mM NaCl or more. At the higher salt concentrations, fibrils fell out of solution nearly instantaneously. 

### 3.3. Plaque Formation after Transfer to pH 7 

**Figure 3:**
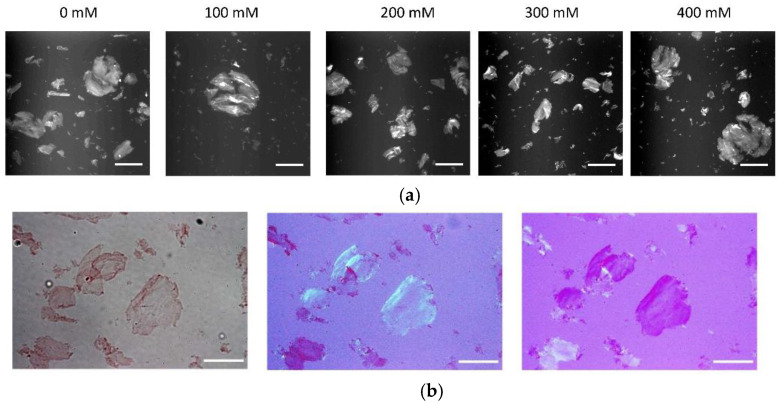
Two-dimensional fibril plaques formed at pH 7. (**a**) Fluorescence images of ThT-stained hewL fibril sheets following transfer to pH 7. pH transfer induces self-assembly into two-dimensionsal fibril plaques at all salt concentrations**.** Scale bar: 200 μm. (**b**) Fibril sheets stained with 50 μM Congo red (left panel) and imaged with crossed polarizers in two slightly different orientations (middle and right). Scale bar: 100 μm.

Next, we diluted 100 μM fibril stock in pH 2 adjusted water five-fold into 50 mM HEPES buffer at pH 7, and at final NaCl concentrations matching those in the above pH 2 experiments. The jump to pH 7 induced precipitation of the fibrils (Figure 3a). Intriguingly, fibril self-assembly switched from the predominant 3-D gel cluster formation at pH 2 to self-assembly into 2D fibril sheets at pH 7. Salt concentration had little to no discernible effect on the propensity of the plaque-like sheets emerging at pH 7, with perhaps a tendency to form smaller sheets in the absence of added NaCl. These sheets readily stained with Congo Red and displayed apple-green birefringence (Figure 3b), which are both commonly observed features of amyloid plaques in tissues. 

### 3.4. Stability of Gels vs. Sheets

**Figure 4:**
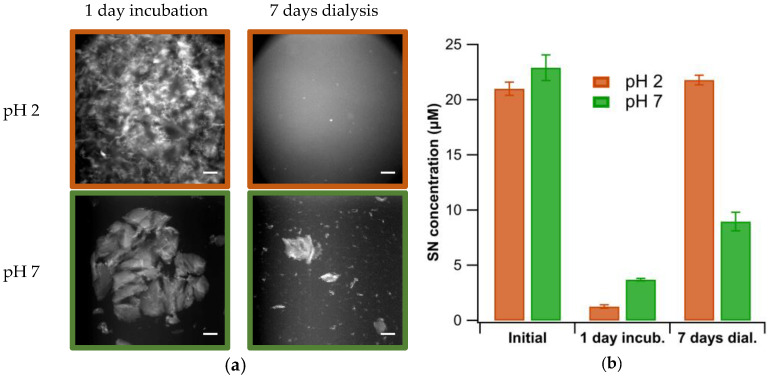
Dissociation of Gels vs. Plaques. (**a**) Images of ThT-stained lysozyme gels and plaques, induced by incubation of isolated fibrils at either pH 2 and 400 mM NaCl (top) or pH 7 and 100 mM NaCl (bottom). Images were taken after 1 day of incubation (left column) and subsequent to 7 days of dialysis at pH 2 and 0 salt (right column). (**b**) Quantification of fibrils in solution following centrifugation at 6000 *g* before incubation, after 1 day of incubation, and following 7 days of dialysis. Scale bar: 100 μm.

We were interested in the stability of the gel clusters formed at pH 2 vs. the fibril sheets emerging at pH 7, upon reversal to the original fibril-stock solution conditions (pH 2, no salt). We induced gelation at pH 2 and plaque formation at pH 7 and allowed the solutions to incubate for 1 day. At that point, the degree of aggregation was determined via centrifugation and the aggregated solutions were dialyzed for 5 days against 25 mM KH_2_PO_4_ buffer at pH 2. As expected, the kinetic aggregation of fibrils into gel clusters was completely reversed upon return to the fibril stock conditions. In contrast, the 2D fibril sheets could only partially be re-dissolved into fibrils over the same dissolution periods (Figure 4a). This is intuitive given the much larger area of mutual contact and, therefore, net attractive interactions among the fibrils in the densely packed fibril sheets compared to the crosslinks in gel clusters. This suggests that the use of 3D amyloid hydrogels, similar to a recent report, might require additional stabilization [48]. The degree of aggregation and subsequent dissolution seen in Figure 4a was also quantified and confirmed using centrifugation (Figure 4b).

## 4. Discussion

The experiments described above document the propensity of isolated amyloid fibrils to assemble into distinct fibrillar suprastructures. This assembly proceeds in the absence of any kinetic growth processes or any extrinsic cellular contributions. Specifically, our results indicate that amyloid fibrils of lysozyme, under some conditions, have the propensity to assemble into disordered 3D gels, consistent with prior results of gel growth from lysozyme monomers [38,39,40]. However, the resulting gel structures do not appear stable against the reversal of solution conditions (Figure 4), which might limit their utility as biomaterials without additional chemical crosslinking [48]. Intriguingly, though, isolated fibrils also readily assembled into 2D fibril sheets, displaying the characteristic apple-green birefringence of amyloid plaques. This seems to replicate the features of amyloid plaques observed in vivo, suggesting that amyloid fibrils do have an intrinsic propensity to form these highly organized supramolecular structures [7,29,49,50]. Fibril assembly into ordered 2D sheets at pH 7 proceeded on a rapid time scale, and often faster than the formation of disordered 3D gels at pH 2 at the lower salt concentrations. The substantial charge repulsion of fibrils at pH 2 is the likely reason for both the stabilization of isolated fibrils in the absence of salt, and for the formation of disordered gel-like structures upon increasing salt concentration. 

Our data do not allow us to determine the microscopic structure of the 3D gels. In particular, we were not able to assess whether gels were fully disordered and whether they formed from individual fibril, pre-assembled fibril bundles, or potentially micro-sheets prior to gelation. There are several indications from this and prior work, though, suggesting that the gel clusters observed at pH 2 represent a disordered gelation process that is qualitatively distinct from 2D plaque formation. For one, the ease with which gel clusters re-dissolved vs. the stability of 2-D sheets (Figure 4) suggests that the former are structurally distinct from the latter. In addition, the reversibility of lysozyme gel formation matches the typical properties of disordered hydrogels. Prior work on lysozyme gels, albeit grown from monomers and under different solution conditions, indicated that the lysozyme formed disordered fibril gels [49]. However, we cannot exclude the possibility that hewL fibrils undergo some modest fibril bundling prior to gelation [50]. 

These observations raise the question, though, what causes the observed switch in the propensity of fibrils to form these two distinct supramolecular assemblies upon changes in salt screening and pH? Since the salt concentrations used at both pH values were identical, the corresponding changes in the Debye screening lengths λ_D_ from about 1 nm (100 mM NaCl) down to 0.5 nm (400 mM NaCl) were identical, as well. Therefore, changes in diffusive charge screening alone do not explain the observed changes in assembly behavior. Changing the pH from 2 to 7 does decrease the net charge of lysozyme monomers from +16 down to +8 charges [47]. The fact that fibrils at pH 2 and the highest salt concentrations of 400 mM can form isolated plaques (Figure 2b,c) suggests that the onset of plaque formation requires a substantial reduction in the net charge repulsion among individual fibrils. This can be accomplished by reducing the fibril net charge itself (pH 7), or by increasing diffusive charge screening well below the monomer radius of about 2 nm.

This reduction in fibril net repulsion, in turn, must reveal an underlying radial anisotropy in the attractive fibril–fibril interactions that promotes the 2D alignment of fibrils over hundreds of micrometers (Figure 3b,c). Ideally, one would like to determine the distribution of surface charges and hydrophobic patches of the fibrils using high-resolution structures of lysozyme amyloid fibrils, which will indicate how individual monomers are rearranged within that structure. As far as we are aware, no such data are available for lysozyme amyloid fibrils. Given the universal propensity of amyloid fibrils to self-assemble into plaques, though, we hypothesize that this anisotropy emerges independent of specific proteins, and as a generic feature of the amyloid fold itself.

Our results show that growth of individual amyloid fibrils can be separated from their subsequent assembly into supramolecular structures. This should significantly broaden the range of amyloid proteins available to generate non-toxic supramolecular structures with amyloid fibrils. In addition, our observations imply that amyloid fibrils, on their own, are capable of assembly into distinct supramolecular structures, widening their appeal to generate multiple distinct biomaterials using the same fibrils as the starting point.

## Data Availability

Data availability upon request.
